# Characterization of the complete mitogenome of Indian Mouse Deer, *Moschiola indica* (Artiodactyla: Tragulidae) and its evolutionary significance

**DOI:** 10.1038/s41598-018-20946-5

**Published:** 2018-02-09

**Authors:** Rama K. Sarvani, Drashti R. Parmar, Wajeeda Tabasum, Neelima Thota, Ara Sreenivas, Ajay Gaur

**Affiliations:** Laboratory for Conservation of Endangered Species (LaCONES), CSIR-Centre for Cellular and Molecular Biology (CCMB) Annexe 1, Hyderguda, Attapur, Hyderabad, 500048 India

## Abstract

The mitochondrial genome of Indian mouse deer (*Moschiola indica*) was sequenced, assembled and characterized for the first time using 22 pairs of polymerase chain reaction (PCR) primers. The mitogenome of *M*. *indica* which is 16,444 bp in size was found very similar to most vertebrates in organisation that harbours 13 protein-coding genes, 22 transfer RNA, 2 ribosomal RNA and 1A + T-rich region. Its comparison with over 52 mitogenomes of the order Artiodactyla, showed a conserved nature of gene organisation, codon usage, gene orientation and evolutionary rates of proteins except that *M. indica* possesses an extra copy of *trnF*. The complete mitogenome and protein-coding genes of *M. indica* were found to be highly A + T biased. Rate of protein evolution was highest in *atp8* and lowest in *cox3*. Further, a higher purifying selection pressure was found to be acting on family Tragulidae compared to Bovidae and Cervidae. The phylogenetic analysis of *M*. *indica* placed the Tragulidae as sister-group of all other ruminants, similar to previous analyses. *Moschiola* forms the sister-group to the other two tragulid genera *Tragulus* (from Asia) and *Hyemoschus* (from Africa), which is unexpected as usually the Asian species are thought to form a monophyletic group.

## Introduction

Most animal mitogenomes are circular and show conserved gene content. The approximate size of the complete mitogenome is 16 kb, encoding 37 genes that comprise 13 protein-coding genes (PCGs), 22 transfer RNA (tRNA) genes, two ribosomal RNA (rRNA) genes and an A + T rich region (control region, CR)^1,2^. A typical mitogenome is characterized by high abundance in each cell, high evolutionary rates, a small genome size, conserved gene content, maternal inheritance and lack of extensive recombination^3–6^. The systematic investigation and comparison of the mitogenome and its distinctive features allow this molecule to be widely used for studying population genetics, evolutionary relationships, phylogenetic relationships and phylogeography in many groups^6–10^.

The Ruminantia underwent a rapid radiation during the Miocene and Pliocene periods, with many new species appearing and many species disappearing. Today, it is one of the most diverse groups in the order Cetartiodactyla, comprising 200 living ruminant species distributed across all continents except Australia and Antarctica^11,12^.

Morphological, molecular and paleontological studies show that tragulids represent the basal branch in the phylogenetic tree of Ruminantia, forming the sister group of Pecora^13–16^. Among the tragulids, the late Eocene Asian ruminant, *Archaeotragulus krabiensis* (Genus: *Archaeotragulus*) was considered the most basal one^17^, representing the only recorded member of the family in Palaeocene period. In the Early Miocene, tragulids were present with a diverse fossil record in Africa, Asia and Europe^18–24^.

The tragulid fossil record includes a number of extinct members but only three genera survived to the present day: *Tragulus* (South East Asia, six species), *Hyemoschus* (Africa, one species), and *Moschiola* (India and Sri Lanka, three species)^25,26^. All tragulids do not possess any cranial appendages and both sexes possess enlarged upper canines. The tragulids were considered as the most primitive animals of all living ruminant families with very little evolutionary history^27^ due to its simple social behaviour, lack of a true omasum^28^, possession of various skeletal structures (e.g., short, unfused metapodials) and retention of an appendix and a gallbladder^27^. The ancestral nature of Tragulidae^13^ has been recently questioned^29^. They belong to the smallest living ungulates and survive as relics in the old world tropical belt^12,23,30,31^.

The genus *Moschiola* (spotted chevrotains) is found in India (*M. indica*) and Sri Lanka (*M. meminna* and *M. kathygre*)^32^. Traditionally, the Asian genera *Moschiola* and *Tragulus* form a monophyletic group with *Hyemoschus* as a sister taxon^23,33^.

Although, the Indian mouse deer is classified as “least concern” on the Red list of International Union for Conservation of Nature (2017), the current population is declining due to poaching. Besides, it is recognised as Schedule I animal in Indian Wildlife Protection Act (1972) as they are heavily hunted for skin and meat for pot.

Studies on molecular and evolutionary aspects of *M. indica* are lacking. A recent study used mitochondrial 12 S rRNA (437 bp) sequence and provided a tool for species differentiation using PCR based-RFLP markers^34^. Previous studies on the karyotype evolution of *Tragulus javanicus* showed that multiple rearrangements took place, most of which appeared to be apomorphic and were not observed in the pecoran (higher ruminants) species. The mouse deer had a low rate of chromosome evolution (0.4 R/Mya) with an approximately similar rate of chromosome changes (1.2 R/Mya) from Cetartiodactyla to Ruminantia and from Ruminantia to Pecora^35^.

The mouse deer are of great importance due to their primitive characters which have not changed much from the Miocene time and would help in understanding the evolution of tragulids. Mitochondrial sequences have been extensively used to resolve the phylogenetic position across many artiodactyls^36–40^, sometimes in combination with nuclear sequences^41,42^.

Hence, the new mitogenome sequence presented here is expected to further provide a lead in to future studies of evolutionary genetics and biogeography of *M*. *indica*. A phylogenetic study of *M*. *indica* would help in designing specific strategies for conservation breeding of this endangered and evolutionary important species. Therefore, the aims of this study were to: (a) generate the first sequence of the complete mitochondrial genome of the Indian tragulid species, *Moschiola indica*. (b) characterize the complete mitogenome of *M. indica* in comparison with other artiodactyls; and (c) investigate the molecular phylogenetics of the species to reaffirm its taxonomic position among Tragulidae.

## Results and Discussion

### Genome structure, organization and composition

**Figure 1:**
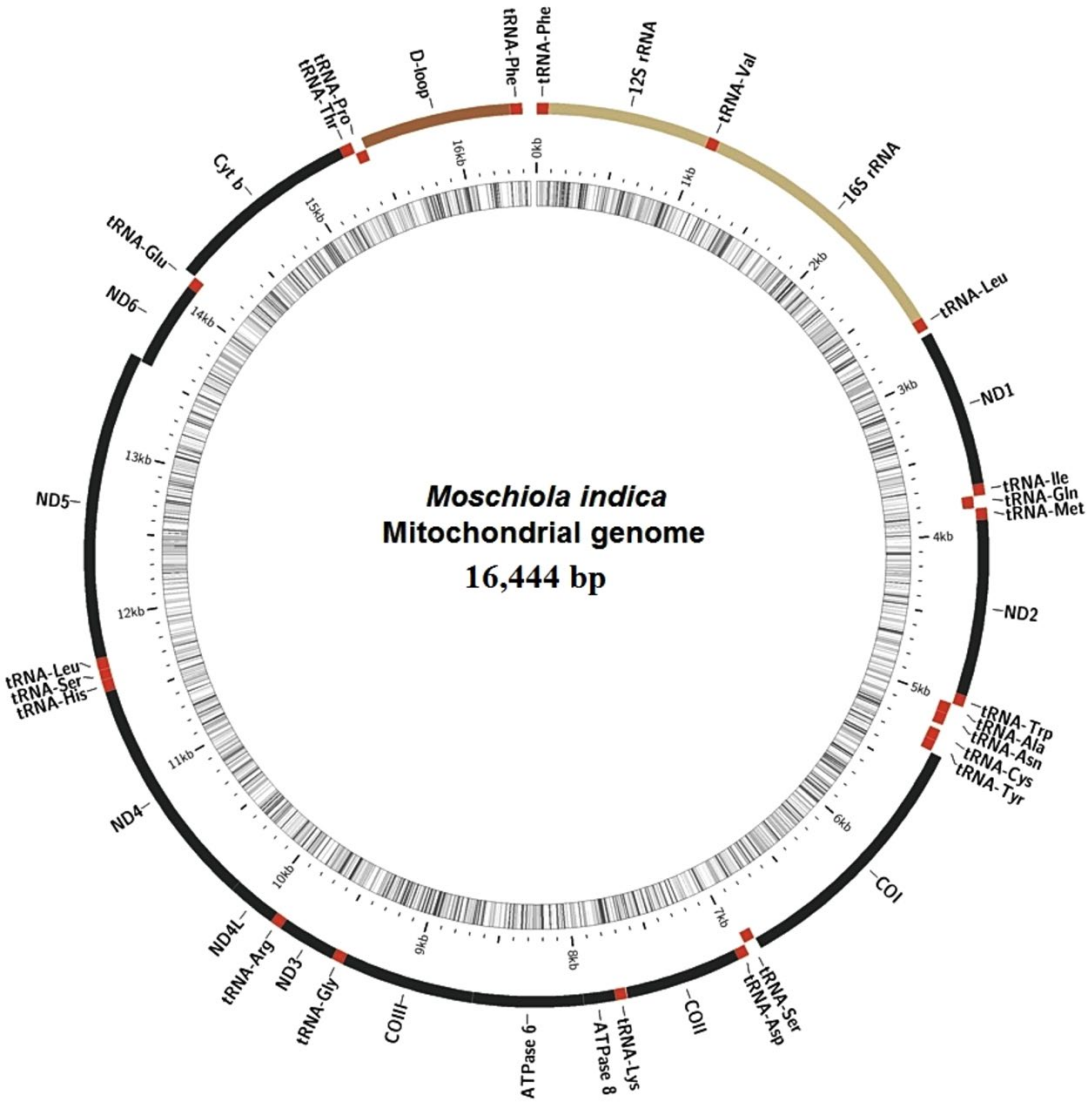
The complete mitochondrial genome organization of *M. indica*. Transfer RNAs (tRNA) are labelled with their corresponding amino acids and are shown in red; *COI*, *COII* and *COIII* refer to subunits of cytochrome c oxidase; *Cyt b* refers to cytochrome b; 12S rRNA and 16S rRNA refer to ribosomal RNAs; *ND1-ND6* refer to components of NADH dehydrogenase; *ATPase 6* and *ATPase 8* refers to classes of ATP synthase.

This paper reports the first complete mitochondrial genome of the Indian mouse deer (*Moschiola indica*), consisting of 16,444 bp (Fig. 1), which is bigger in size than the mitogenome of two other studied tragulid species i.e. South East Asian, *Tragulus kanchil* (16,333 bp) and African, *Hyemoschus aquaticus* (16,225 bp). The complete mitogenome of *M. indica* encodes a total of 37 genes, out of which 13 were protein-coding genes (PCGs), 22 transfer RNA, 2 ribosomal RNA genes and an A + T rich region (Table 1), which is typically observed in vertebrates. The novel mitogenome sequence of *M. indica* was deposited in GenBank with the accession number KY290452.

**Table 1:** The organization and characterization of the complete mitochondrial genome of *M. indica*.

Gene	Strand	Location	Size	Anticodon	Intergenic Nucleotides
*tRNAF* ^*Phe*^	H	1–70	70	GAA	0
*12S rRNA*	H	71–1028	958	—	0
*tRNAV* ^*Val*^	H	1029–1094	66	TAC	−2
*16S rRNA*	H	1093–2668	1576	—	2
*tRNAL2* ^*Leu*^	H	2671–2744	74	TAA	8
*nad1*	H	2753–3694	942	—	8
*tRNAI* ^*Ile*^	H	3703–3771	69	GAT	−3
*tRNAQ* ^*Gln*^	L	3769–3841	73	TTG	2
*tRNAM* ^*Met*^	H	3844–3912	69	CAT	0
*nad2*	H	3913–4941	1029	—	13
*tRNAW* ^*Trp*^	H	4955–5021	67	TCA	1
*tRNAA* ^*Ala*^	L	5023–5090	68	TGC	0
*tRNAN* ^*Asn*^	L	5091–5163	73	GTT	32
*tRNAC* ^*Cys*^	L	5196–5264	69	GCA	0
*tRNAY* ^*Tyr*^	L	5265–5331	67	GTA	7
*cox1*	H	5339–6877	1539	—	7
*tRNAS2* ^*Ser*^	L	6885–6953	69	TGA	6
*tRNAD* ^*Asp*^	H	6960–7026	67	GTC	0
*cox2*	H	7027–7707	681	—	6
*tRNAK* ^*Lys*^	H	7714–7779	66	TTT	1
*atp8*	H	7781–7978	198	—	−37
*atp6*	H	7942–8616	675	—	5
*cox3*	H	8622–9404	783	—	1
*tRNAG* ^*Gly*^	H	9406–9474	69	TCC	0
*nad3*	H	9475–9819	345	—	2
*tRNAR* ^*Arg*^	H	9822–9889	68	TCG	0
*nad4L*	H	9890–10183	294	—	−4
*nad4*	H	10180–11547	1368	—	10
*tRNAH* ^*His*^	H	11558–11628	71	GTG	0
*tRNAS1* ^*Ser*^	H	11629–11688	60	GCT	0
*tRNAL1* ^*Leu*^	H	11689–11758	70	TAG	0
*nad5*	H	11759–13561	1803	—	7
*nad6*	L	13569–14087	519	—	3
*tRNAE* ^*Glu*^	L	14091–14159	69	TTC	4
*cob*	H	14164–15297	1134	—	6
*tRNAT* ^*Thr*^	H	15304–15376	73	TGT	−1
*tRNAP* ^*Pro*^	L	15376–15442	67	TGG	0
A + T rich Region	—	15443–16332	890	—	0
*tRNAF* ^*Phe*^	H	16333–16402	70	GAA	—

The total coverage of each groups of genes in the mitogenome of *M. indica* was as follows: 13 PCGs (73.3%), 22 tRNA genes (9.8%), and 2 rRNA genes (16.4%). In order to determine the exact position and orientation of genes in the mitochodrium of *M*. *indica* with reference to other previously studied tragulids, the complete mitogenome of *M. indica* was compared to publically available data of *T. kanchil* and *H. aquaticus* as well as other members of the order Artiodactyla. Although, the gene order and gene orientation in the mitochondrial genome of *M. indica* was overall similar among all the members of Artiodactyla, we found some notable differences in positions and lengths of few genes as well as a gene duplication event in *M. indica* in comparison of the other two tragulids. Almost all the genes in the mitogenome of *M. indica* were located on the H strand except *nad6* and eight tRNAs (*tRNA*^*Gln*^*, tRNA*^*Ala*^, *tRNA*^*Asn*^*, tRNA*^*Cys*^*, tRNA*^*Tyr*^*, tRNA*^*Ser*^, *tRNA*^*Glu*^, *tRNA*^*Pro*^), which were found to be located on the L strand.

### Base composition and skewness

AT-skew, GC-skew, and A + T content are parameters that are frequently used to investigate the pattern of nucleotide composition of mitochondrial genomes^43,44^. Altogether a high A + T content (61.4%) was observed in complete mitogenome of *M. indica*, similar to other artiodactyls (Table 2), the highest A + T content being observed in *trnR* (77.9%).

**Table 2:** Nucleotide composition indices in various regions of nine representative mitogenomes of artiodactyls. ^a^Hiendleder *et al*. 2008^81^, ^b^Yang *et al*.^37^, ^c^Hassanin *et al*.^36^, ^d^Cho *et al*. 2016^82^, ^e^Ji *et al*. 2009^83^.

Species	Family	Accession number	Whole		Protein Coding Genes (PCGs)		Large Ribosomal RNA (*rrnl*)		Small Ribosomal RNA (*rrns*)	
			Length (bp)	AT (%)	Length (bp)	AT (%)	Length (bp)	AT (%)	Length (bp)	AT (%)
*Bos indicus*	Bovidae	AF492350^a^	16,339	60.5	11,313	60.0	1571	61.3	956	59.0
*Moschus chrysogaster*	Moschidae	JQ608470^b^	16,353	62.1	11,319	61.8	1572	62.4	955	59.7
*Axis axis*	Cervidae	NC_020680^c^	16,349	62.8	11,310	62.9	1571	62.7	955	60.3
*Giraffa camelopardalis*	Giraffidae	JN632645^c^	16,433	60.4	11,319	59.9	1575	61.6	956	59.7
*Moschiola indica*	Tragulidae	KY290452 (This Study)	16,444	61.4	11,310	61.2	1576	61.7	958	56.4
*Hyemoschus aquaticus*	Tragulidae	NC_020714^c^	16,225	59.1	11,304	58.4	1578	61.4	957	58.8
*Tragulus kanchil*	Tragulidae	NC_020753^c^	16,333	58.8	11,292	58.1	1575	59.6	958	56.4
*Sus scrofa*	Suidae	AY574047^d^	16,651	60.5	11,301	60.3	1572	62.7	963	59.6
*Camelus bactrianus*	Camelidae	NC_009628^e^	16,659	58.1	11,319	57.8	1567	61.5	968	57.1

**Figure 2:**
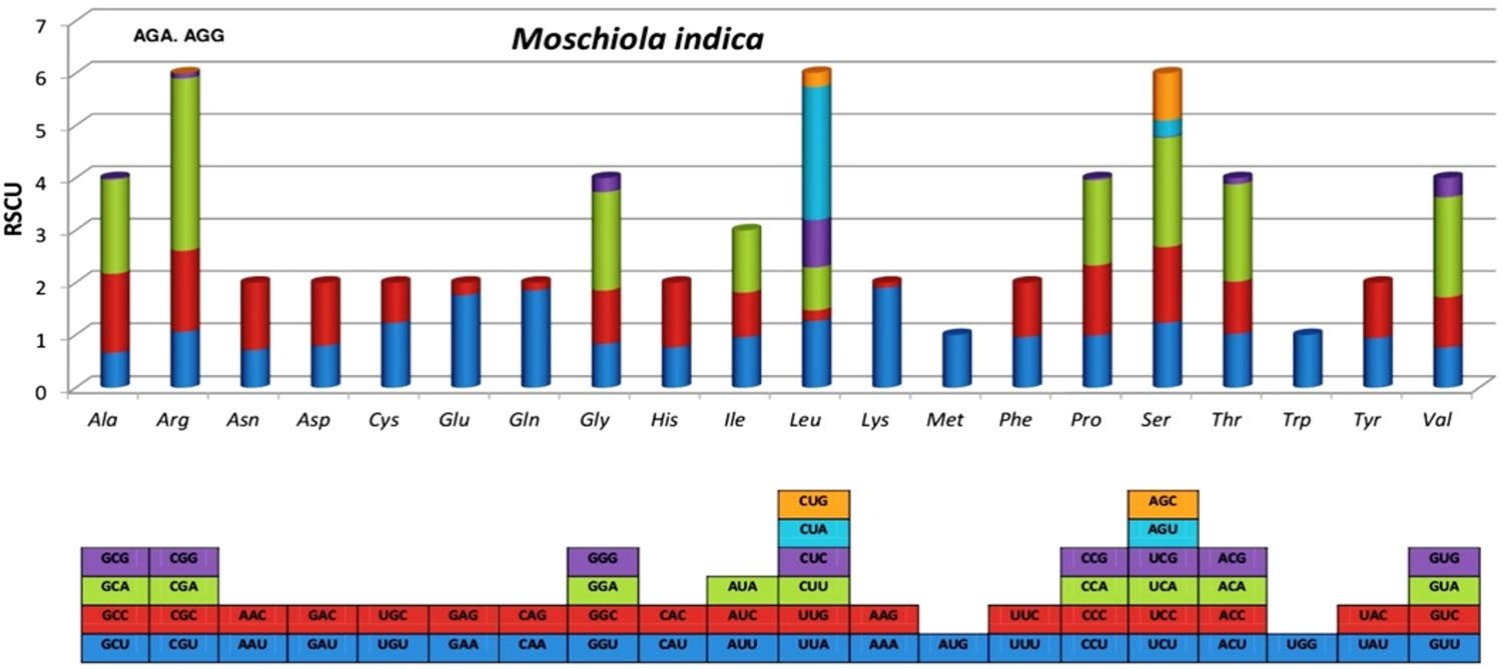
The Relative Synonymous Codon Usage (RSCU) of the mitochondrial protein-coding genes of *M*. *indica*. Different codons present in PCGs are plotted on X axis. Codons which are not present in mitogenome are indicated above the bar.

**Figure 3:**
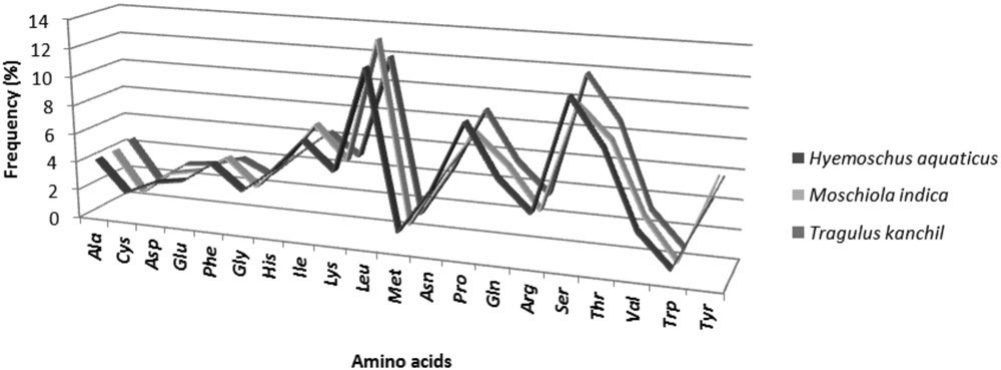
Amino acid composition and their relative frequency (%) in complete mitogenome of *Moschiola indica*, *Tragulus kanchil* and *Hyemoschus aquaticus* of the family Tragulidae.

A significant bias towards A/T was observed in the codon usage of the mitochondrial genomes of *M. indica* (Fig. 2), as also observed in other artiodactyls. The amino acid distribution and their relative frequencies were quite similar among the three species representing the genera of Tragulidae family i.e. *M. indica*, *T. kanchil* and *H. aquaticus* (Fig. 3). The most frequent amino acids were *Leu* (11.5–12.8%), *Ser* (9.7–10.4%), *Thr* (8.2–8.6%), *Pro* (7.7–9.2%) and *Ile* (7.5–9.9%), while *Trp* was rare (0.8–1.1%), as seen in other artiodactyls.

### Protein-coding genes and rate of evolution

The total length of concatenated 13 PCGs in the mitogenome of *M. indica* was estimated to be 11,310 bp and accounted for 68.76% of the complete mitogenome. Out of 13 PCGs, 12 were located on the majority strand (H-strand), while *nad6* was located on the minority strand (L-strand), as observed in other artiodactyls. The overall A + T content of 13 PCGs in *M. indica* was 61.2%, ranging from 54.3% (*cox3*) to 66.7% (*atp8*). The concatenated data of 13 PCGs of *M. indica* showed total 9247 (82.0%) variable sites and 7176 (63.7%) parsimony informative sites.

**Figure 4:**
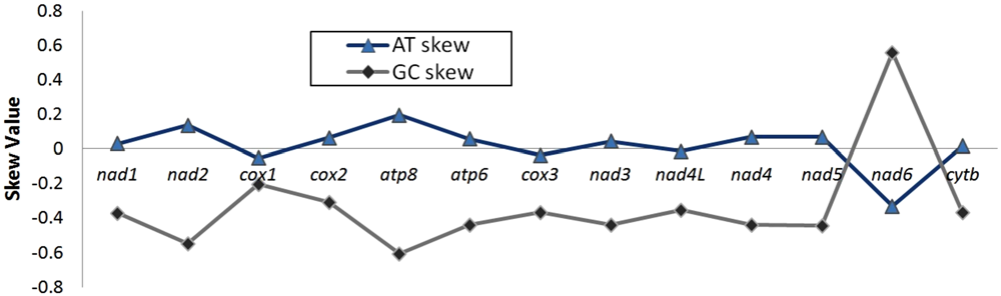
Graphical representation of AT- and GC-skew in all the 13 protein-coding genes of *M*. *indica* mitogenome.

Base skews were estimated in order to understand the degree of base bias between all PCGs. The average AT and GC skew values are shown for the PCGs of *M. indica* in comparison with other artiodactyls studied here (Table 3). Positive AT skewness (0.026) was observed for most of the PCGs, indicating that adenines occur more frequently than thymines, similar to other related species including other two tragulids^36^ (Table 3). Negative GC skewness was observed for most of the PCGs of *M. indica* ranging from −0.203 to −0.604, suggestive of C biased nucleotide composition. A deviation from these ranges in AT skew (−0.331) and GC skews (0.560) were observed in *nad6* region, which was also observed in *T. kanchil* (AT skew = −0.346, GC skew = 0.589) and *H. aquaticus* (AT skew = −0.340, GC skew = 0.622). The trend of AT-skew and GC-skew values in all 13 PCGs of *M. indica* is shown in Fig. 4. Twelve out of 13 PCGs showed notable anti-G bias at third codon position, which is in congruence with other Tragulidae^36^.

**Table 3:** The AT and GC skew in the protein-coding genes of nine representative mitogenomes of artiodactyls used in this study.

Species	Protein Coding Genes (PCGs)					
	T (U)	C	A	G	AT-skew	GC-skew
*Bos indicus*	28.7	26.8	31.3	13.2	0.043	−0.340
*Moschus chrysogaster*	29.8	25.6	32.0	12.6	0.035	−0.340
*Axis axis*	31.2	24.4	31.7	12.8	0.007	−0.311
*Giraffa camelopardalis*	28.7	26.7	31.2	13.5	0.026	−0.328
*Moschiola indica*	29.8	26.2	31.4	12.6	0.026	−0.352
*Hyemoschus aquaticus*	28.6	27.7	29.8	13.9	0.020	−0.330
*Tragulus kanchil*	27.8	28.4	30.3	13.5	0.044	−0.354
*Sus scrofa*	27.4	26.9	32.9	12.8	0.091	−0.355
*Camelus bactrianus*	29.2	27.0	28.6	15.3	−0.042	−0.276

All of the 13 PCGs started with ATN (ATG or ATA: putative start codons), similar to *H. aquaticus* but differed in *nad4l* of *T. kanchil* which started with GTN. A few abnormal start codons were also observed that included GCC (*atp8*), AAA (*cox3* and *nad3*), TTG (*cytb* and *nad5*) and ACC (*nad6*). Five out of thirteen PCGs had complete stop codons i.e. TAN (TAA or TAG). Other five genes (*atp8*, *cox1*, *cox2*, *nad1*, *nad3*) had AGA as a stop codon while two of the genes (*atp6*, *cox3*) had AGG as a stop codon.

The evolutionary dynamics of PCGs among related species can be best estimated by evaluating synonymous (dS) and nonsynonymous (dN) substitution rates^45,46^. To determine the impact of selection pressure on artiodactyls along with tragulids, the relative ratio of dN/dS was estimated for PCGs of nine representative species from each family of artiodactyls (Table 4). The *atp8* gene was found to have the highest evolutionary rate with a dN/dS ratio of 0.2318 (95% CI = 0.1876–0.2831) while *cox3* had the lowest ratio at 0.0218 (95% CI = 0.0173–0.0270) suggestive of a low rate of evolution. Although, the selection pressure for all genes was different, the dN/dS for 13 PCGs were all less than 1 (95% CI), suggestive of the presence of purifying selection in these species. The varying rates of selection pressure among all the functional genes indicated different ways of independent evolution^47^. Moreover, all 13 PCGs of the Tragulidae had altogether higher dN/dS ratio (0.0385959, with 95% CI) than compared to Bovidae (0.0365208, with 95% CI) and Cervidae (0.0370097, with 95% CI) and lower to those of Suidae (0.0462901, with 95% CI) and Camelidae (0.0426647, with 95% CI). These results imply weaker purifying selection at PCGs in Tragulidae than in Bovidae and Cervidae.

**Table 4:** Evolutionary rate estimates in each mitochondrial PCG across mitogenomes of nine representative species of artiodactyls: *B. indicus, M. chrysogaster, A. axis, G. camelopardalis, M. indica, T. kanchil, H. aquaticus, S. scrofa* and *C. bactrianus*. dN/dS refers to the ratio of nonsynonymous substitutions and synonymous substitutions with 95% confidence interval (CI).

Gene	dN/dS (95% CI)
*nad1*	0.0350 (0.0296–0.0411)
*atp6*	0.0487 (0.0412–0.0571)
*atp8*	0.2318 (0.1876–0.2831)
*cox1*	0.0505 (0.0470–0.0543)
*cox2*	0.0434 (0.0384–0.0489)
*cox3*	0.0218 (0.0173–0.0270)
*cytb*	0.0673 (0.0618–0.0734)
*nad2*	0.0841 (0.0773–0.0914)
*nad3*	0.0717 (0.0613–0.0837)
*nad4l*	0.0684 (0.0569–0.0819)
*nad4*	0.0426 (0.0376–0.0479)
*nad5*	0.0993 (0.0937–0.1052)
*nad6*	0.0885 (0.0785–0.0997)

### Ribosomal RNA and transfer RNA genes

The *rrnS* and *rrnL* genes in the mitogenome of *M*. *indica* were positioned between *trnF* and *trnV*, and between *trnV* and *trnL2*, respectively. Both rRNAs were separated by *trnV* which is typically observed in most vertebrates^48^. The length of the *rrnS* and *rrnL* was 958 bp and 1576 bp respectively. Total content of A + T of two rRNA was 59.7% which is in congruence with other two tragulids studied here (58.5% for *T. kanchil* and 60.5% for *H. aquaticus*). The length and A + T content of both rRNAs among all the representative species of artiodactyls were much alike (Table 2).

**Figure 5:**
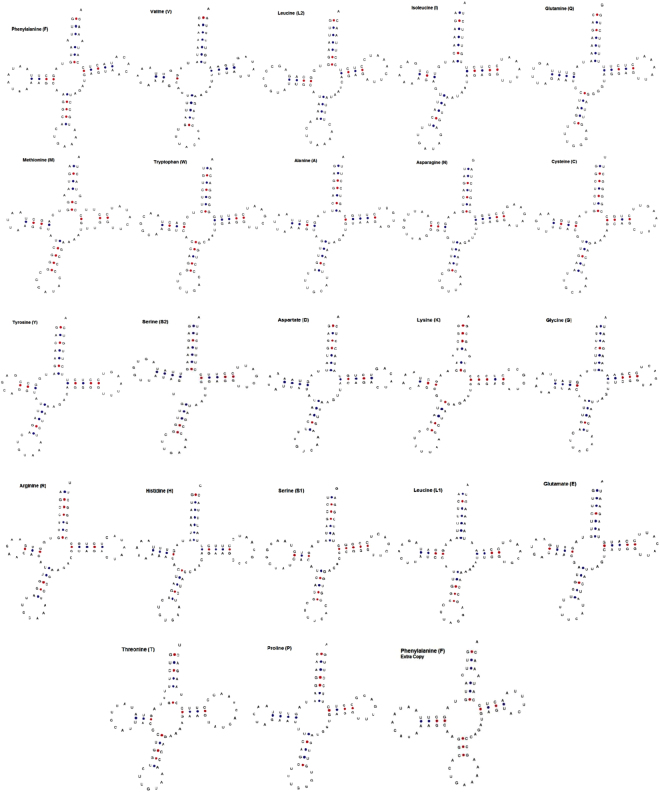
Secondary structures of the 22 tRNA genes of the *M. indica* mitogenome.

Total number of tRNA genes coding for amino acids in mitogenome of *M. indica* was inferred by tRNAscan-SE. The anticodons of all the tRNAs found in the complete mt genome of *M. indica* were identical to other Artiodactyla species. Out of total 22 t-RNA genes, the range of coverage varied from 60 bp (*trnS1*) to 74 bp (*trnL2*). The tRNAs were found to have an average base composition of A: 32.9%, T: 31.4%, G: 19.3% and C: 16.3%, with the highest GC content in *trnK* (53.1%) and the lowest in *trnR* (22.1%). Out of 22 tRNAs, 14 genes were located on H strand while others were located on L strand (Table 1). All the tRNA could be folded in to a secondary clover-leaf structure (Fig. 5) as predicted by Mitos WebServer^49^. Apart from the classic secondary base pair structure of tRNA i.e. A-U and C-G, total ten mismatched base pairs were found in seven tRNAs of *M. indica* mitogenome. The type of mismatch varied on different stems for all seven mismatched pairs of tRNAs where seven were in the amino acid acceptor stems, two in the pseudouridine (TΨC) stems and one in anticodon stem (Table 5).

**Table 5:** The details of the mismatched t-RNA base pairs from *M. indica*. AA = amino acid acceptor, TΨC = pseudouridine, AC = anticodon.

tRNA	Mismatched base pairs	Stem	Frequency
*trnF*	A-A	AA	1
	A-C	AA	1
*trnM*	U-U	TΨC	2
	A-G	AA	1
*trnW*	A-C	AC	1
*trnK*	A-G	AA	1
*trnR*	A-C	AA	1
*trnS1*	A-C	AA	1
*trnT*	U-U	AA	1

### Overlapping and intergenic spacer regions

In complete mitogenome of *M. indica*, five sequences were found overlapped with a total length of 47 bp ranging from 1 bp to 37 bp of length. The longest overlap was observed between *atp8* and *atp6* (37 bp), being highest between the same genes of the other two tragulids studied (34 bp for both *T. kanchil* and *H. aquaticus*). Besides, overlap was observed between *trnT* and *trnP* (1 bp); between *trnV* and *rrnL* (2 bp); between *trnI* and *trnQ* (3 bp); and between *nad4l* and *nad4* (4 bp). This long (34–37 bp) and short (4 bp) overlap of the two PCGs, between *atp8 and atp6* and between *nad4l* and *nad4* respectively which were located on the H strand, is typically observed in most species of artiodactyls.

The intergenic spacers of *M. indica* mitogenome were observed at almost 20 regions ranging from 1 bp to 32 bp, amounting to a total of 131 bp in length (Table 1). The longest spacer (32 bp) was found between *trnN* and *trnC* and was highly rich in A + T content. This long spacer region was typically observed in all artiodactyl families except camelidae where the length of this spacer was 33 bp. Overall, intergenic spacers in *M. indica* were longer than when compared to both *T. kanchil* (124 bp over 18 regions) and *H. aquaticus* (114 bp over 19 regions).

### The A + T-rich region

The 890 bp (15,443–16,332 nt), non-coding A + T-rich control region was organized between *trnP* and *trnF* genes. The length of this region for the representative species of artiodactyls were in the range of 700–1,300 bp which is typical among mitochondrial genomes of vertebrates^50^. This region is longer in *M. indica* than found in *T. kanchil* (827 bp), *H. aquaticus* (789 bp), *G. camelopardalis* (727 bp), and *A. axis* (687 bp), but shorter than in *B. indicus* (911 bp), *M. chrysogaster* (923 bp), *S. scrofa* (1173 bp) and *C. bactrianus* (1247 bp). The higher size variation in control region (CR) than other regions of mitogenome is the reflection of multiple tandem repeats (TR) and differences in their copy numbers^51^. The total A + T content, AT skew and GC skew in this region was 63.4%, 0.012 and −0.322 respectively. No noticeable long repeats were found in CR of *M. indica*. In particular, 26 bp repeat consensus (GTACATATTATTATTTATAGTACATA) harbouring within 15608–15658 bp was found twice at 3′ portion of CR. No similar motif was present in any other artiodactyls’ species except in *T. kanchil* which was present at similar positions indicating occurrence of the duplication events before the species diverged.

### Duplications and palindromes

In comparison with the putative ancestral gene arrangement of Artiodactyla, there seems to be at least one rearrangement event in the mitogenome of *M. indica*: an extra *trnF* like structure on H strand immediately following the CR and spanning the length of 70 bp (16333–16402 bp), similar to the one observed at the beginning of the complete mitochondrial structure (1–70 bp) of *M. indica*. This *trnF* like structure was unique to *M. indica* and not observed in any other species of artiodactyls. Moreover, total eight nucleotide substitutions and two gaps were found between the two *trnF* sequences including four synonymous and four non synonymous substitutions. A similar result has been observed in other artiodactyls, i.e. a unique *rrnS* like structure immediately after the CR in *Peccari tajacu* of Suidae family^36^. These duplicated regions surrounding the origin of replication are the spots of major rearrangement events as strand slippage and inaccurate termination include duplicated blocks of genes^6,52^. However, a re-validation of this characterization is suggested.

A single palindromic sequence 5′-CTTCTCCCGCC-3′ (11 bp) was consistently observed between 5163–5173 bp range in all artiodactyl species studied except in the Suidae.

### Phylogenetic relationship

**Figure 6:**
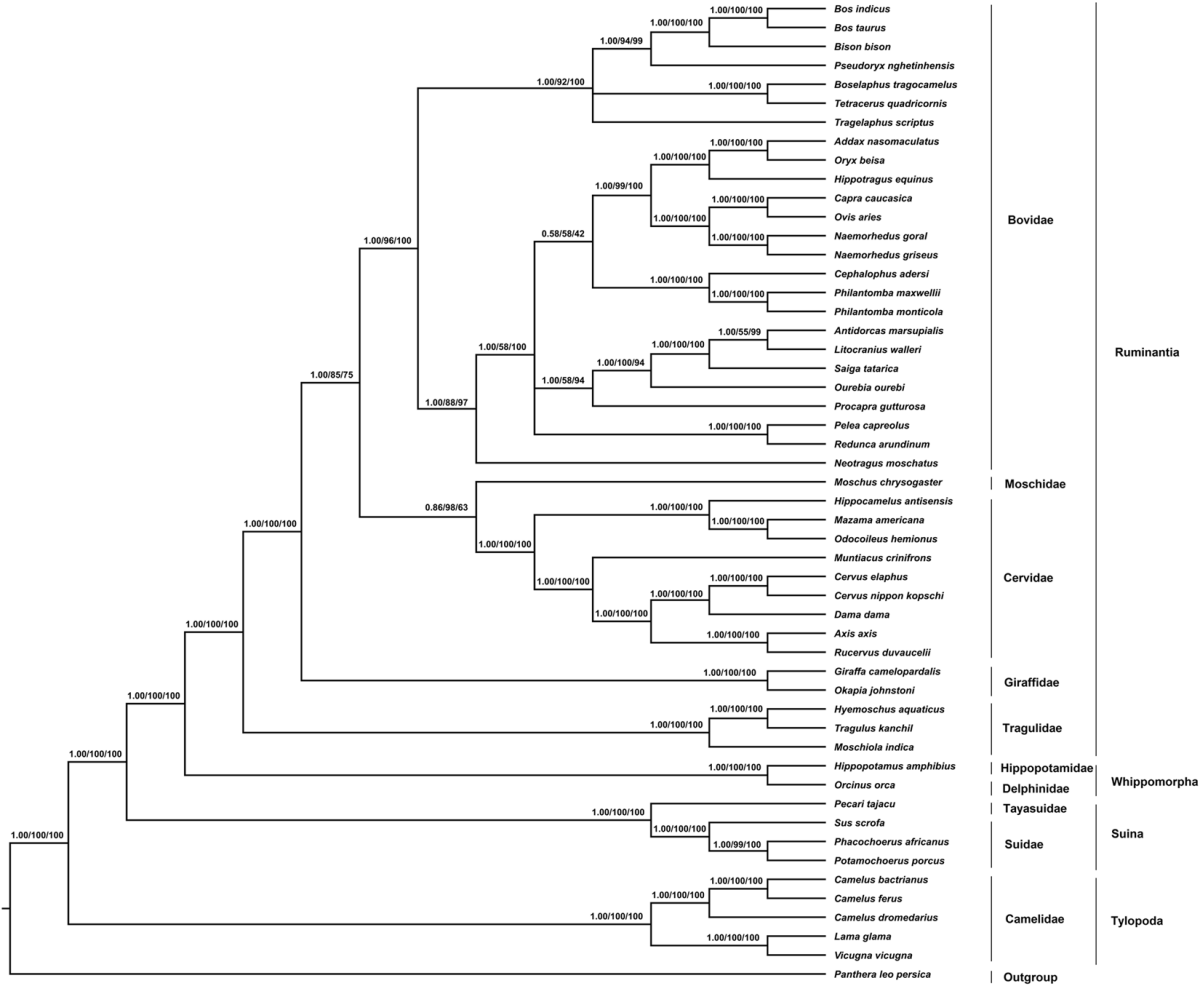
Phylogenetic relationship among 52 mitogenomes of Artiodacyla, reconstructed from concatenated sequences of 13 PCGs using Bayesian inference (BI) and Maximum Likelihood (ML) methods. At each node, the values follow in this order: Bayesian Posterior Probability (BPP) done by MrBayes v3.2.5/Bootstrap value for ML analyses done by MEGA 6.06/Bootstrap value for ML analyses done by raxmlGUI v1.3.

We provide a fully resolved phylogeny of Artiodactyla, including one or multiple representatives from each major group (Fig. 6). For Bayesian and ML analyses, we used concatenated sequences of 13 PCGs from 52 artiodactyls species. The tree topology of the ruminant sub-tree was consistent in both BI and ML analysis with high posterior probability (>0.95) and bootstrap support (>70), respectively. Besides, no significant changes in the topology of the trees were observed when comparing the results of BI and ML analysis using complete mitogenome of all 52 species of Artiodactyla. The closest living relatives of Ruminantia, an ancodontan (Hippopotamidae) and a cetacean (Delphinidae) were used to root the ingroup of Pecora + Tragulina. The entire Cetartiodactyla tree was rooted with a Pantherinae species i.e. *Panthera leo persica* and the resultant topology was consistent with the topology obtained from previous studies^39,41,53,54^. The Tragulidae was placed as the sister group to all other ruminants, which is in congruence with Hassanin *et al*.^36^ and Bibi^16^. Although, the relative position of Bovidae, Cervidae and Moschidae were not consistent with previous studies^39,41,54,55^, the present study revealed Cervidae and Moschidae forming a sister clade to Bovidae^33,53^. Other than relative position of Cervidae, Bovidae and Moschidae, our analysis strongly supports the relationship among the ruminants as previously described in other studies^33,36,41,53,56^. No earlier evidences of the molecular studies including more than two living species of Tragulidae have been found except the study done by Agnarsson and May Collado in 2008^54^ where the *Tragulus* and *Hyemoschus* formed a distinct clade in the family Tragulidae with *Moschiola meminna* nested within Bovidae making both families (Tragulidae and Bovidae) paraphyletic. The probable reason for such ambiguity observed in the position of *Moschiola* might be the use of only mitochondrial cytochrome b sequence shorter than 30% for phylogenetic study. Contrary to the previous studies^33^, where *Hyemoschus* was the sister group to the Asian tragulids, our BI and ML analysis strongly support the placement of *Moschiola* as the sister group to the other tragulid genera *Tragulus* + *Hyemoschus* with highest posterior probability (1.00) and maximum bootstrap support (100%), respectively.

Tragulidae was first to diverge among other ruminants forming a basal branch^13,14,41,57–59^, which was confirmed in the present study using molecular data with strong nodal support (posterior probability [PP] = 1.00 and bootstrap proportion [BP] = 100).

This report is the first molecular characterization of complete mitogenome of Indian tragulid species i.e. *M. indica*. The phylogenetic position of *M. indica* in the family Tragulidae holds importance as it is considered to be the evolutionary link between the families of Artiodactyla. Although, the complete mitogenome of *M*. *indica* showed similar characters with other Artiodactyla species, it differed from other tragulids by the events of duplications. The analysis of selection pressure in 13 PCGs of Tragulidae suggested accumulation of slightly more beneficial nonsynonymous mutations. The characterization of the complete mitogenome and distinctness of the Indian tragulid species from the other two genera using molecular data would propagate further studies on the biogeography of the species, evolution of the genes and to address other evolutionary linkages among this extraordinary family Tragulidae, and other Artiodactyla species.

## Materials and Methods

### Sample Collection and DNA extraction

Post-mortem tissues of four animals housed in the Nehru Zoological Park, Hyderabad were obtained opportunistically, in full compliance with permission of the Central Zoo Authority of India. Tissues were stored in ethanol at 4 °C until DNA extraction. High molecular weight DNA was extracted from samples using Phenol-chloroform method^60^. Total genomic DNA was dissolved in TE buffer (10 mM Tris, 0.1 mM EDTA). The extracted DNA was quantified using NanoDrop® ND-1000 spectrophotometer (NanoDrop Technologies, Inc. Wilmington, DE, USA) followed by 0.8% agarose gel electrophoresis for checking the integrity of DNA. Isolated DNA was stored at −20 °C until further use.

### PCR amplification and sequencing

PCR amplification of mitochondrial markers was carried out in 15 µl reaction mixture containing 40 ng/µl of genomic DNA, 10 × PCR buffer, 10 mM dNTPs, 1.5 mM MgCl_2_, 5 pM of each primer and 0.5 units of Amplitaq gold (Applied Biosystems, USA). All 22 primers used in this study are listed in Supplementary Table SI. The following PCR conditions were used: initial denaturation at 95 °C for 7 min, denaturation at 94 °C for 45 s; annealing at specific Tm for 50 s and extension at 72 °C for 1 min 20 s (40 cycles) with final extension at 72 °C for 7 mins. PCR products were separated in 1.5% agarose gel using electrophoresis. All the amplified products were sequenced using 3730 DNA Analyser (ABI, USA).

### Sequence alignment and complete mitogenome annotation

The complete mitogenome sequence data was assembled and analyzed using Seqman program of Lasergene software^61^. Mitochondrial DNA annotation was done using Mitos WebServer^49^ and MitoFish^62^. MitoAnnotator^62^ was used to generate a gene map of complete mitogenome of *M. indica*. Careful manual annotation was conducted using the Artemis software^63^ with the help of BLAST, for ensuring the gene boundaries^64^. The transfer RNA (t-RNA) predictions and their secondary structures were confirmed using tRNAscan-SE software^65^ and Mitos WebServer^49^. Sequence alignment with their related species’ homologs was performed for the t-RNAs that could not be identified with the above two approaches. The r-RNAs, PCGs and control region were identified by comparing with other artiodactyl mitogenomes.

For the comparative sequence analysis with other Artiodactyla including Tragulidae, complete mitochondrial sequences of one or many representatives from each major group of Artiodactyla were downloaded from the National Centre for Biotechnology Information (NCBI) database (Accession numbers are given in Supplementary Table SII). These sequences were aligned with the generated *M. indica* sequence in MEGA 6.06^66^ using ClustalW^67^ and the aligned sequences were used for comparative gene characterization and phylogenetic tree re-construction.

The nucleotide sequences of the PCGs were translated using mtDNA genetic code of other vertebrates. ClustalX 2.0^68^ was used for identification of exact start codons and stop codons of all putative amino acid sequences. Nucleotide (A + T content) and amino acid compositions were estimated and compared for all the three representatives of Tragulidae and other representative species from Artiodactyla using MEGA 6.06. To estimate the bias in nucleotide composition among the genes of the complete mitogenome of *M. indica*, AT and GC skew values were determined following the established method^69^: AT-skew = (A − T)/(A + T) and GC-skew = (G − T)/(G + T). The intergenic spacer regions and overlapping regions between genes of complete mitogenome of *M. indica* were determined manually.

The values of Relative Synonymous Codon Usage (RSCU) of the complete mitogenome of *M. indica* were calculated using MEGA 6.06. Datamonkey Webserver^70^ of HyPhy package^71^ was used to estimate synonymous substitutions per synonymous sites (dS) and nonsynonymous substitutions per nonsynonymous sites (dN) for all 13 PCGs of each representative species from artiodactyls. The SLAC^72^ method with 95% confidence interval was applied for all the nine species to estimate dN/dS bias. The complete mitogenome sequence was examined for possible tandem repeats as well as palindromes using Tandem Repeats Finder 4.0^73^ and EMBOSS software suite^74^, respectively.

### Phylogenetic Analysis

To ascertain molecular based phylogenetic position of *M*. *indica* and its relationship with other Artiodactyla, analysis with Bayesian Inference (BI) method using MrBayes v3.2.5^75^ and Maximum Likelihood (ML) method using raxmlGUI v1.3^76^ interface as well as MEGA 6.06 was performed on 13 PCGs of these 52 species’ sequence alignments. The accession numbers, mitogenome sizes and taxonomic information of total 52 species of Artiodactyla are provided in Supplementary Table SII. For the purpose of comparative topology study with 13 PCGs of one or more representative species of Artiodactyla, we also performed complete mitogenome phylogeny with BI and ML methods.

*Panthera leo persica* (KU234271)^77^ was used as an outgroup. The thirteen concatenated nucleotide sequences of PCGs were aligned with MEGA 6.06. For the phylogenetic analysis, the resulting aligned sequences of each gene were concatenated forming a single contig of 11,322 bp. For each PCG genes, the best-fit nucleotide substitution model was selected using adjusted parameters (gapped regions were included) in jModelTest 2.1.5^78,79^. Sequences failing to align along the length of the core domain (and therefore containing potential sequencing/splicing artifacts) were excluded. According to the BIC (Bayesian Information Criterion), GTR + I + G was selected as a best-fit model for all the concatenated genes except *cox1*, *atp 6* and *cytb* genes where HKY + I + G substitution model, *atp 8* where HKY + G substitution model and *nad 6* where GTR + G substitution model were selected as a best fit model.

With 10 million generations initiated from a random tree, we performed two separate runs with four different Markov Chain Monte Carlo (MCMC) chains which sampled one tree every 1000 generations. To assess the convergence of the BI analyses for all the parameters, we used potential scale reduction factors (PSRF) near to 1.0 and the average standard deviation of split frequencies below 0.01. Tracer v1.6^80^ was used to scrutinize the convergence of the BI analyses. A total of 200202 number of trees in two separate runs were generated to obtain the final consensus tree, of which total of 150152 trees were sampled (each run having 100101 trees, of which 75076 number of trees sampled). As conservation burn-in, the first 25% of the tress were discarded. Bayesian posterior probability (BPP) values were used as estimation for the BI tree support. For ML analysis in raxmlGUI v1.3^76^ and in MEGA 6.06, we employed GTR + I + G substitution model for each concatenated gene. The bootstrap analysis of 1,000 iterations provided a measure of confidence for the detected relationships.

## Electronic supplementary material

Supplementary Files
