# Supplementary material for: Characterization of the complete mitogenome of Indian Mouse Deer, Moschiola indica (Artiodactyla: Tragulidae) and its evolutionary significance
Source: Sci Rep. 2018 Feb 9;8:2697. doi: 10.1038/s41598-018-20946-5 (PMC5807545; doi:10.1038/s41598-018-20946-5)
Supplement: Supplementary file 1 — Supplementary Files [file 41598_2018_20946_MOESM1_ESM.pdf]

Characterization of the complete mitogenome of the Indian Mouse Deer, *Moschiola indica* (Artiodactyla: Tragulidae) and its evolutionary significance

Rama K. Sarvani[#], Drashti R. Parmar[#], Wajeeda Tabasum, Neelima Thota, Ara Sreenivas, Ajay Gaur*

Laboratory for Conservation of Endangered Species (LaCONES), CSIR-Centre for Cellular and Molecular Biology (CCMB) Annexe 1, Hyderguda, Attapur, Hyderabad 500048 India

*agaur@ccmb.res.in, Telephone: +91-40-24006433, FAX No.: +91-40-24006441

[#] These authors contributed equally to this work

Supplementary I. Details of published primers used to amplify the complete mitogenome of *M. indica*[1]

S.No	Primers	Primer sequence (5'→3')	TM (℃)
1	DLU405	ACC ATG CCG CGT GAA ACC AGC A	56.5
2	12SL41	GYG YGG ATR CTT GCA TGT GTA	
3	12SU829	GCA CGC ACA CAC CGC CCG TCA C	56.5
4	16SL518	CGC TTT CTT AAT TGR TGG CTG C	
5	16SU365	AGC CTG GTG ATA GCT GGT TGT CC	58.1
6	16SL1056	AAG CTC CAT AGG GTC TTC TCG TC	
7	16SU946	CCG TGC AAA GGT AGC ATA ATC A	54.9
8	N1L64	CCT AGN ACT TTT CGT TCN ACT A	
9	Uleu	GTG GCA GAG CCC GGT AAT TG	52
10	IleL	TTACTCTATCAAAGTAACTC	
11	N1U840	TYC GAG CAT CHT AYC CHC GAT T	52
12	N2L492	TGG TTT AGB CCB CCT CAK CCY CC	
13	N2U354	CAC TTY TGA GTN CCA GAA GT	56.7
14	AsnL	TAG GGT RTT TAG CTG TTA AC	
15	TrpU	AGA CCA AGA GCC TTC AAA GC	50
16	C1L339	GCT TCW ACT ATD GAD GAT GC	
17	C1U246	GGN GGN TTY GGH AAY TGA CT	57.9
18	C1L1017	GAA RAT RAA GCC TAG RGC TCA	
19	C1U897	TTY ACH GTH GGA ATA GAY GTC	50
20	C2L15	GCR TCT TGR AAN CCT ART TG	
21	SerU	CCC CCY AYW RYT GGT TTC AAG CCA	55.4
22	A8L1	GTK GAY GTR TCT AGT TGY GGC AT	
23	C2U603	CAA TGC TCH GAR ATY TGY GG	55.4
24	C3L45	GAN ARD GCT CCY GTD AGN GGT CA	
25	A6U654	GCC TAY GTN TTY ACY CTN CTA GT	50
26	GlyL	TGA TTG GAA GTC ARY TGT AC	
27	C3U780	GTH TCY ATC TAT TGA TGA GG	57.9
28	N4L27	CAG GTY AGR GGD ATD AGT AT	
29	U213M1	AGC YTG YGA AGC AGC ACT AGG	51.7
30	L918M1	GCK GTR GCT CCT ATR TAR CTT CA	
31	N4U840	AGC TCH ATY TGY YTH CGY CAA AC	51.7
32	Leu2L	CCA ATT TTT TGG YTC CTA AGR CC	
33	Ser2U	CCG AAA AAG YAY GCA AGA ACT GC	51.7
34	N5L652	GCD GAT TTT CCD GTK GCD GCT A	
35	N5U1	GAC GAR CAG AYG CHA AYA CAG C	56.7
36	N5L1214	GTD AKT ADD AGG GCT CAG GCG	
37	N5U1146	GGM AGC CTN GCN YTA ACA GG	56
38	N6RL154	AGT TTA ATG GDH TDG GDG ATT G	
39	N6RU102	CCA TAA CTR TAY AAA GCH GCA A	50
40	CBL402	CCT CAR AAT GAT ATT TGK CCT CA	
41	CBU162	CAG GMC TAT TCC TRG CHA TAC A	50
42	LTHR	CCC TTY TCT GGT TTA CAA GAC C	
43	U1068	CAT CGG ACA ACT AGC ATC TAT	52
44	L482	CCT GAA GWA AGA ACC AGA TG.	

Supplementary II. A list of mitogenomes used for comparative characterization and phylogenetic analysis, name of species, size of the mitogenome, family, accession numbers and reference.

Species	Size of the mitogenome (bp)	Family	Accession no.	Reference
Vicugna vicugna	16,084	Camelidae	NC_013558	2
Camelus bactrianus	16,659	Camelidae	NC_009628	3
Camelus dromedarius	16,643	Camelidae	NC_009849	Huang *et al.*, 2009 (GenBank-NCBI)
Camelus ferus	16,680	Camelidae	NC_009629	3
Lama glama	16,597	Camelidae	NC_012102	Yasue *et al.*, 2009 (GenBank-NCBI)
Phacochoerus africanus	16,719	Suidae	DQ409327	4
Potamochoerus porcus	16,693	Suidae	NC_020737	5
Sus scrofa	16,651	Suidae	AY574047	Cho *et al.*, 2016 (GenBank-NCBI)
Pecari tajacu	16,818	Tayasuidae	JN632683	5
Moschiola indica	16,444	Tragulidae	KY290452	This Study
Hyemoschus aquaticus	16,225	Tragulidae	JN632650	5
Tragulus kanchil	16,333	Tragulidae	JN632709	5
Giraffa camelopardalis	16,433	Giraffidae	JN632645	5
Okapia johnstoni	16,422	Giraffidae	NC_020730	5
Addax nasomaculatus	16,751	Bovidae	NC_020674	5
Antidorcas marsupialis	16,444	Bovidae	NC_020678	5
Bison bison	16,321	Bovidae	GU946999	6
Bos indicus	16,339	Bovidae	AF492350.1	7
Bos taurus	16,339	Bovidae	GU947021	6
Boselaphus tragocamelus	16,322	Bovidae	EF536350	5
Capra caucasica	16,624	Bovidae	NC_020683	5

Cephalophus adersi	16,435	Bovidae	NC_020685	5
Hippotragus equinus	16,436	Bovidae	JN632647	5
Litocranius walleri	16,438	Bovidae	JN632653	5
Naemorhedus goral	16,555	Bovidae	JX188255	8
Naemorhedus griseus	16,448	Bovidae	NC_020723	5
Neotragus moschatus	16,399	Bovidae	NC_020728	5
Oryx beisa	16,518	Bovidae	NC_020793	5
Ourebia ourebi	16,392	Bovidae	NC_020733	5
Ovis aries	16,617	Bovidae	KF302458	9
Pelea capreolus	16,589	Bovidae	NC_020734	5
Philantomba maxwellii	16,440	Bovidae	NC_020735	5
Philantomba monticola	16,451	Bovidae	NC_020736	5
Procapra gutturosa	16,546	Bovidae	NC_020738	5
Pseudoryx nghetinhensis	16,358	Bovidae	NC_020616	5
Redunca arundinum	16,439	Bovidae	NC_020794	5
Saiga tatarica	16,375	Bovidae	NC_020746	5
Tetracerus quadricornis	16,343	Bovidae	NC_020788	5
Tragelaphus scriptus	16,402	Bovidae	NC_020751	5
Moschus chrysogaster	16,353	Moschidae	JQ608470	8
Cervus nippon kopschi	16,429	Cervidae	HQ832482	10
Dama dama	16,330	Cervidae	NC_020700	5
Axis axis	16,349	Cervidae	NC_020680	5
Hippocamelus antisensis	16,410	Cervidae	JN632646	5
Mazama americana	16,478	Cervidae	NC_020719	5
Muntiacus crinifrons	16,357	Cervidae	AY239042	Li *et al.*, 2016

				(GenBank-NCBI)
Cervus elaphus	16,357	Cervidae	NC_007704	Wada *et al.*, 2010 (GenBank-NCBI)
Odocoileus hemionus	16,482	Cervidae	NC_020729	5
Rucervus duvaucelii	16,342	Cervidae	NC_020743	5
Orcinus orca	16,386	Delphinidae	GU187219	11
Hippopotamus amphibius	16,402	Hippopotamidae	AP003425	Yasue *et al.*, 2016 (GenBank-NCBI)
Panthera leo persica	17,059	Pantherinae (Outgroup)	KU234271	12

List of References:

1. Hassanin, A., Ropiquet, A., Couloux, A. & Cruaud, C. Evolution of the Mitochondrial Genome in Mammals Living at High Altitude: New Insights from a Study of the Tribe Caprini (Bovidae, Antilopinae). *J. Mol. Evol.* **68,** 293-310 (2009).
2. Di Rocco, F., Zambelli, A., Maté, L. & Vidal-Rioja, L. The complete mitochondrial DNA sequence of the guanaco (*Lama guanicoe*): comparative analysis with the vicuña (*Vicugna vicugna*) genome. *Genetica*. **138,** 813-818 (2010).
3. Ji, R. *et al.* Monophyletic origin of domestic bactrian camel (*Camelus bactrianus*) and its evolutionary relationship with the extant wild camel (*Camelus bactrianus ferus*). *Anim. Genet.* **40,** 377-82 (2009).
4. Wu, G. S. *et al.* Population phylogenomic analysis of mitochondrial DNA in wild boars and domestic pigs revealed multiple domestication events in East Asia. *Genome Biol.* **8,** R245 (2007).
5. Hassanin, A. *et al.* Pattern and timing of diversification of cetartiodactyla (mammalia, laurasiatheria), as revealed by a comprehensive analysis of mitochondrial genomes. *C. R. Biol.* **335,** 32-50 (2012).

6. Douglas, K. C. *et al.* Complete mitochondrial DNA sequence analysis of *Bison bison* and bison-cattle hybrids: function and phylogeny. *Mitochondrion*. **11,** 166–175 (2011).
7. Hiendleder, S., Lewalski, H. & Janke, A. Complete mitochondrial genomes of *Bos taurus* and *Bos indicus* provide new insights into intra-species variation, taxonomy and domestication. *Cytogenet. Genome. Res.* **120,** 150-156 (2008).
8. Yang, C., Xiang, C., Zhang, X. & Yue, B. The complete mitochondrial genome of the Alpine musk deer (*Moschus chrysogaster*). *Mitochondrial DNA*. **24,** 501-503 (2013).
9. Lancioni, H. *et al.* Phylogenetic relationships of three Italian Merino-derived sheep breeds evaluated through a complete mitogenome analysis. *PLoS ONE*. **8,** e73712 (2013).
10. Pan, H. C., Fang, H. Y., Jin, C. & Liu, L. The complete mitochondrial genome of sika deer *Cervus nippon* (Cetartiodactyla: Cervinae) South Anhui population. *Mitochondrial DNA*. **25,** 85-86 (2014).
11. Morin, P. A. *et al.* Complete mitochondrial genome phylogeographic analysis of killer whales (*Orcinus orca*) indicates multiple species. *Genome Res.* **20,** 908-916 (2010).
12. Tabasum, W., Ara, S., Rai, N., Thangaraj, K. & Gaur, A. Complete mitochondrial genome sequence of Asiatic lion (*Panthera leo persica*). *Mitochondrial DNA*. **1**, 619-620 (2016).
